# Genetic analysis of the *Staphylococcus epidermidis *Macromolecular Synthesis Operon: Serp1129 is an ATP binding protein and *sigA *transcription is regulated by both σ^A^- and σ^B^-dependent promoters

**DOI:** 10.1186/1471-2180-10-8

**Published:** 2010-01-12

**Authors:** Kendall A Bryant, Lauren C Kinkead, Marilynn A Larson, Steven H Hinrichs, Paul D Fey

**Affiliations:** 1 Department of Pathology and Microbiology, University of Nebraska Medical Center, Omaha, NE. 68198-5900 USA

## Abstract

**Background:**

The highly conserved macromolecular synthesis operon (MMSO) contains both *dnaG *(primase) and *sigA *(primary sigma factor). However, in previously evaluated gram-positive species, the MMSO is divergent upstream of *dnaG*. The MMSO of *Bacillus subtilis *contains three open reading frames (ORFs) that are differentially regulated by multiple promoters. In conjunction with studies to determine the expression profile of *dnaG*, the MMSO of *Staphylococus epidermidis *was characterized.

**Results:**

The ORFs of *S. epidermidis *were compared to the previously described MMSO of *B. subtilis *and two additional ORFs in *S. epidermidis, serp1129 *and *serp1130*, were identified. The largest transcript, 4.8 kb in length, was expressed only in exponential growth and encompassed all four ORFs (*serp1130, serp1129, dnaG*, and *sigA*). A separate transcript (1.5 kb) comprising *serp1130 *and *serp1129 *was expressed in early exponential growth. Two smaller transcripts 1.3 and 1.2 kb in size were detected with a *sigA probe *in both exponential and post-exponential phases of growth. Western blot analysis correlated with the transcriptional profile and demonstrated that Serp1129 was detected only in the exponential phase of growth. Computational analysis identified that Serp1130 contained a CBS motif whereas Serp1129 contained an ATP/GTP binding motif. Functional studies of Serp1129 demonstrated that it was capable of binding both ATP and GTP. Comparisons with a *sigB:dhfr *mutant revealed that the 1.3 kb *sigA *transcript was regulated by a σ^B^-dependent promoter.

**Conclusions:**

These studies demonstrated that the *S. epidermidis *1457 MMSO contains two ORFs (*serp1129 *and *serp1130*) not described within the *B. subtilis *MMSO and at least three promoters, one of which is σ^β^-dependent. The transcriptional regulation of *sigA *by σ^B ^provides evidence that the staphylococcal σ^B^-dependent response is controlled at both the transcriptional and post-transcriptional level. The conservation of *serp1129 *across multiple gram-positive organisms and its capability to bind ATP and GTP support the need for further investigation of its role in bacterial growth.

## Background

Replication of the bacterial chromosome is a complex process requiring the interaction of a variety of essential enzymes including primase, helicase, and DNA polymerases I and III [1]. It is hypothesized that bacteria co-regulate the expression of these unlinked genes to ensure the necessary proteins are available during chromosomal replication. To better understand these processes, the transcriptional regulation of the macromolecular synthesis operon (MMSO) [2], which contains *dnaG *(primase), was studied in *Staphylococcus epidermidis*.

The MMSO was originally identified in *Escherichia coli *where it was found to consist of three genes with seemingly divergent functions; *rpsU, dnaG*, and *rpoD *[3]. The first open reading frame (ORF), *rpsU*, encodes an essential S21 ribosomal protein whereas the second (*dnaG*) encodes primase, an enzyme required to synthesize RNA primers during DNA replication. The third ORF (*rpoD*) encodes the primary sigma factor (σ^A^) responsible for promoter recognition by RNA polymerase [3-5]. Investigations of other bacteria determined that the structure and composition of the MMSO was conserved in multiple gram-negative species and *rpoD *(*sigA *in gram-positive bacteria) and *dnaG *are linked [2]. The most well characterized gram-positive MMSO is that of *Bacillus subtilis *which closely resembles the *E. coli *MMSO. The only exception is the 5' end where an uncharacterized gene, *yqxD*, is found instead of an *rpsU *ortholog [6-8]. Within the *B. subtilis *MMSO, there are seven known promoters; two σ^A^-dependent, two σ^H^-dependent, one σ^B^-dependent, and two promoters for which the sigma factor involved is unknown [9]. Since *S. epidermidis *is a non-spore forming bacteria, contains only four known sigma factors (σ^A^, σ^B^, σ^S ^and σ^H^) [10-13], and has a divergent genetic organization upstream of *dnaG*, we hypothesized that the transcriptional regulation of the *S. epidermidis *MMSO would differ from *B. subtilis*. Our study found the *S. epidermidis *MMSO consists of four genes (*serp1130, serp1129, dnaG*, and *sigA*) and is regulated by at least three distinct promoters. In addition, it was determined that two promoters, one of which is σ^B^-dependent, regulate *sigA *transcription suggesting that the staphylococcal σ^B ^response is tempered by the enhancement of *sigA *transcription. Finally, functional studies demonstrated that Serp1129 was an ATP/GTP binding protein.

## Methods

### Growth of bacterial strains

All time course studies were performed with *S. epidermidis *strains 1457 [14] and 1457 *sigB::dhfr *[15]. Overnight cultures of the bacteria were used to inoculate flasks of tryptic soy broth (TSB; Becton-Dickinson) to an OD_600 _of 0.1 which corresponds to the 0 time point of the growth curve. The strains were grown aerobically (10:1 flask:volume ratio; 250 rpm) in TSB at 37°C.

### Isolation of RNA

The bacteria were grown as described above. Samples of the cultures were harvested at 2 hour intervals and processed using a combination of the FastPrep FP120 (Bio 101) and the RNeasy kit (QIAGEN) as recommended by the manufacturer's protocol and Roberts et al. [16].

### Northern blot and RT-PCR analysis

A 1% (wt/vol) agarose (Sigma) gel containing 0.66 M formaldehyde and morpholinepropanesulfonic acid (MOPS) running buffer (20 mM MOPS, 10 mM sodium acetate, 2 mM EDTA; pH 7.0) was used to separate 5 μg of total RNA. The RNA was then transferred to a positively charged nylon membrane (Roche) by overnight capillary transfer in 20× SSC (0.3 M Na_3_-Citrate, 3.0 M NaCl; pH 7.0). Double stranded DNA probes were constructed using the PCR DIG Probe Synthesis Kit (Roche) according to the manufacturer's recommendations. The *serp1130, serp1129, dnaG *and *sigA *probes were amplified using primers 1035/1036, 672/673, 942/943, and 674/675 respectively (Table 1). RNA probes were constructed by first cloning the *S. epidermidis *1457 *sigA *gene (using primers 674 and 675; Table 1) into the PCR cloning vector pCR2.1 (Invitrogen). The *sigA *gene was subsequently digested from pCR2.1 using *HindIII *and *XbaI *and cloned into pSPT18 (Roche). Sense and anti-sense RNA was transcribed and labeled with digoxygenin using both the SP6 and T7 promoters as described by the manufacturer (Roche). The subsequent hybridization and development of the blots were performed as described by the manufacturer's DIG manual (Roche). Molecular weights were estimated using an RNA molecular weight marker 0.5-10 kb (Invitrogen).

**Table 1:** Primers used in study.

Primer Number	5'-3' Sequence	Description
942	GCATTTGAAGCGGGTTTGTTATC	Forward *dnaG*
943	ATTGACTAAAGAAAGGTCTC	Reverse *dnaG*
1035	GGCTATACTCACGATGTCTGG	Forward *serp1130*/RT-PCR primer
1036	TTTATATTATCCATGTACTTACCACC	Reverse *serp1130*
670	ACATGTGTTTATTTACTTTTCAACCCGC	RT-PCR primer
672	ATTGTTGCCTCTGATTCAATTGGTG	Forward *serp1129*/RT-PCR primer
673	TGCATATCTAGCTTTGTCACCTAATCC	Reverse *serp1129*/RT-PCR primer
675	TTTAACAATTCGTATGCTGCATGG	Reverse *sigA*
760	CTTGCTTTCGAATAAATTTACGTGC	RT-PCR primer
674	AAGCAAACAATTGATCCGACTTTAACAC	RT-PCR primer/forward *sigA*
677	GGATTTTATATCACGCAGTGCTTCTAAC	RT-PCR primer
940	ATTATTATCAAAAAATTGTGCC	RT-PCR primer
1135	TTTAAATACATACGCACTGGGTC	RT-PCR primer
1178	CATCGTGAGTATAGCCAAGTCAGGACG	Primer extension 5' end
1196	TCAGCTATATGCTCGCCCGTGATAG	Primer extension 5' end
1194	CTTGGTTGTCAGACATGAAAAGGCCT	Primer extension 3' end
1211	AGGTGTTTTTGATGTTCTTGAAATGCC	Primer extension
1212	TTCGATAACAATTCCATTAGTGGACCC	Primer extension
1213	GATTTGCAGCATAAATAACGAGTTCCTG	Primer extension
1214	ATCTTCTAAAAAGATGGTACATATAGC	Primer extension
1215	ATCTATACGCAAAAGACATCACCTATC	Primer extension
1222	AAGTGATATTACCTTAAATAAGCGG	Primer extension 3' end
1224	AATCACTAGATTACACTGAGTAAGTG	Primer extension 3' end
1319	TAATTCTGAATCTCCAATACG	GSP*1 at 3' end of *dnaG *for 5' RACE
1320	TTTGAATATAGTCCTCTATTTCG	GSP*2 at 3' end of *dnaG *for 5' RACE
1321	TAACTAAATCTAATATATCAG	GSP*1 at 5' end of *dnaG *for 5' RACE
1322	TTTATTTCATCAATGACGGATTG	GSP*2 at 5' end of *dnaG *GSP2 for 5' RACE
731	GTAAGT*CCATGG*CATGGATAATATAAAGATAATTG	Forward *serp1129 *cloning primer with *NcoI *site
732	GATCA*GGATCC*GGTTGCTAAAAGAATGAAGG	Reverse *serp1129 *cloning primer with *BamHI *site

*GSP = Gene specific primer

Italics in primers 731 and 732 represent the *Nco*I and *Bam*HI restriction sites, respectively.

RT-PCR was performed using a One Tube RT-PCR kit (Roche) according to the manufacturer's recommendations. Oligonucleotides used for detection of specific genes as indicated in text are listed in Table 1. All reactions were allowed to proceed for 22 cycles with an annealing temperature of 50°C. Amplified products were visualized by ethidium bromide staining after separation on a 1.5% agarose gel (Sigma).

### Primer extension analysis

Primers were end-labeled with γ-^32^P 4500 mCi/mmol (MP Biomedical) using T4 kinase (Promega). The primers used to detect the +1 transcriptional start site 5' to *serp1130 *and *sigA *were 1178/1196 and 1194/1222/1224, respectively (Table 1). Reverse transcriptase (RT) reactions were performed using Monster Script 1^st ^Strand cDNA Synthesis Kit (Epicenter) and 40 pmol of end-labeled primers annealed to 10 μg of total RNA. The RT reactions were ethanol precipitated and resuspended in 3 μl of water and 3 μl of loading buffer (95% formamide, 10 mM EDTA, 0.1% Xylene Cyanol and 0.1% Bromophenol Blue [final pH 11]). A sequencing ladder was synthesized using SequiTherm EXCEL II DNA Sequencing Kit (Epicenter). The sequencing ladder was loaded beside the RT samples and separated on a 6% denaturing acrylamide sequencing gel (6% acrylamide/Bis 19:1, 6 M urea, 1× TBE).

### 5' Race Analysis

5' Race analysis was performed using the 5' RACE System, Version 2.0 (Invitrogen) as recommended by the manufacturer with primers 1319 through 1322 as indicated in Table 1.

### Construction of plasmid for expression of recombinant *S. epidermidis *Serp1129

The open reading frame of *S. epidermidis serp1129 *was amplified using primers 731 and 732 that contained an *Nco*I and *BamH*I restriction sites, respectively. The resulting 962 bp product was then digested with *BamH*I and *Nco*I and ligated into the *BamH*I and *Nco*I sites of pET30a+ vector (Novagen). The resulting plasmid (pNF174) was electroporated into *E. coli *BL21-DE3 (Novagen) for protein production. The plasmid sequence was verified by sequencing in both directions by the University of Nebraska Medical Center (UNMC) Eppley Molecular Biology Core Facility.

### Expression and Purification of *S. epidermidis *Serp1129

*E. coli *BL21(DE3) containing pNF174 was grown (shaken at 250 rpm; 37°C) in 1 L of 2xYT media containing 30 μg kanamycin per mL. At an OD_600 _of 0.6, the culture was induced with 0.5 mM of IPTG (isopropyl-β-D-thiogalactopyranoside; Sigma) and grown (shaken at 250 rpm) for an additional 2 hours at 25°C. Cultures were pelleted by centrifugation at 5,000 × *g *for 15 min at 4°C and the cell pellets were resuspended in 100 ml of binding buffer (50 mM Tris, 30 mM imidazole, 500 mM NaCl pH 7.4). Cells were lysed by 4 passages through an EmulsiFlex (Avestin, Inc.). Proteases were inhibited by the addition of 0.4 mM phenylmethylsulfonyl fluoride (PMSF). Soluble cell extracts were obtained by centrifugation at 12,000 × *g *for 30 min at 4°C. The lysates were applied to a HisTrap HP column (GE Healthcare) at a flow rate of 0.5 ml/min. After binding, the column was washed with 20 column volumes of binding buffer. The purified Serp1129 was eluted with elution buffer (50 mM Tris, 500 mM imidazole, 500 mM NaCl pH 7.4). Finally, elution fractions containing Serp1129 were dialyzed against 50 mM Tris (pH 7.5). The dialyzed sample was then frozen at -80°C.

### Detection of Serp1129

*S. epidermidis *was grown as described above and total protein was extracted at 2, 4, 6, 8, 10, and 12 hours as follows. The bacteria were pelleted by centrifugation at 3,000 × g and resuspended in 1 ml TDS buffer (10 mM NaPO_4_, 1% Triton X v/v, 0.5% Deoxycholate w/v, 0.1% SDS w/v) containing 0.4 mM PMSF. The cells were lysed by the addition of 50 μg lysostaphin followed by incubation at 37°C for 30 min. Cellular DNA was sheared by passage through a 40-gauge needle four times and digested with 10 μg DNaseI at 37°C for 30 min. The total protein lysates were then concentrated using Microcon Ultracel YM-10 concentrators (Millipore). A 10% SDS-PAGE was loaded with 40 μg of total protein extract from each time point and subsequently transferred to an Immobilon-P Transfer membrane (Millipore) by electroblotting at 200 mAmp for 90 minutes. The membrane was first blocked in TBST (100 mM Tris 0.9% NaCl and 0.1% Tween 20) containing 10% skim milk, and subsequently incubated with a 1:1000 dilution of the anti-Serp1129 antibody (see below) diluted in TBST. A 1:1000 dilution (in TBST) of alkaline-phosphatase conjugated AffiniPure goat anti-mouse IgG (Jackson ImmunoResearch) was incubated with the membrane for an additional hour. The blot was developed using 10 mL of NBT/BCIP (Roche) as recommended by the manufacturer. A Serp1129 monoclonal antibody was produced by the UNMC Monoclonal Antibody Laboratory using the peptide sequence GKDPKGLPKADIVLLGIC as an antigen. A final cysteine residue was added for coupling adjuvants.

### ATP/GTP Binding Assay

The ATP/GTP binding reaction consisted of 1 μg of recombinant Serp1129 and 1 μM of Adenosine 5' triphosphate [γ] azidoanilide 2', 3'-Biotin or 1 μM of Guanosine 5' triphosphate [γ] azidoanilide 2', 3'-Biotin (Affinity Labeling Technologies). The 20 μl reaction was incubated for 30 seconds at 25°C and then crosslinked by UV irradiation at 4,000 μW/cm^2 ^at 254 nm. Reactions containing 5, 10, 20, and 30 μM of unlabeled ATP or GTP were performed as described above. The samples were placed in SDS-PAGE loading buffer, boiled for 5 min, separated by10% SDS-PAGE electrophoresis, and then transferred to Immobilon-P Transfer membrane (Millipore Corporation) by electroblotting at 200 mA for 100 minutes. The blot was blocked in TBST (100 mM Tris 0.9% NaCl and 0.1% Tween 20) containing 10% skim milk. A 1:8000 dilution of Peroxidase Streptavidin (Jackson ImmunoResearch) was made in TBST and the membrane was incubated at room temperature for 1 hour with shaking. The blot was developed using the ECL Western Blotting Analysis System (GE Healthcare) as recommended by the manufacturer.

## Results

### Genetic organization of the *S. epidermidis *MMSO and other gram-positive bacterial MMSOs

**Figure 1:**
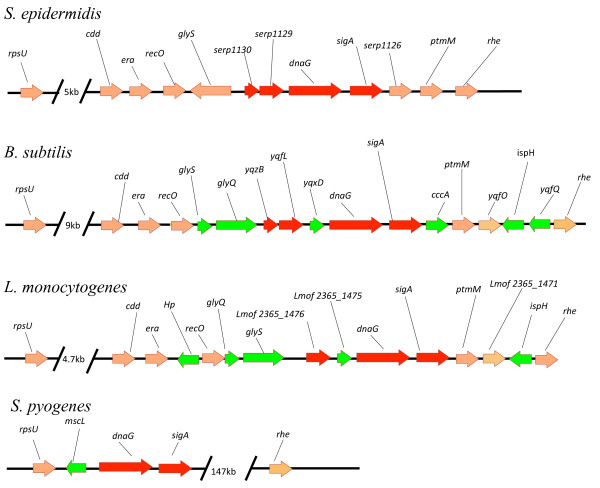
**Schematic diagram demonstrating the conservation of the MMSO region in four gram-positive bacteria**. Genes contained within the *S. epidermidis *MMSO and their equivalents in *Bacillus subtilis, Listeria monocytogenes*, and *Streptococcus pyogenes *are highlighted in red. Orthologues that were identified in *B. subtilis, L. monocytogenes*, or *S. pyogenes *that are not found in *S. epidermidis *(between *rpsU *5' of the MMSO and *rhe *3' of the MMSO) are highlighted in green.

Examination of the *S. epidermidis *RP62A [GenBank CP000029] and ATCC 12228 [GenBank AE015929] genomes revealed that both *dnaG *and *sigA *were linked as previously described, however, structural differences were also apparent in comparison with *B. subtilis *str. 168 [GeneBank AL009126] (Figure 1). The presence of potential new ORFs within the *S. epidermidis *MMSO led us to investigate the degree of conservation of the MMSO region within 2 other gram-positive genomes, *Listeria monocytogenes *str. 4b F2365 [GeneBank AE017262] and *Streptococcus pyogenes *MGAS9429 [GeneBank CP000259] (Figure 1). Several observations were noted when comparing these genomes. First, the *sigA *and *dnaG *genes were linked in all four genomes suggesting the presence of an MMSO. In addition, the genes surrounding the MMSO (in between *rpsU *upstream and *rhe *downstream) were moderately conserved between *S. epidermidis, L. monocytogenes*, and *B. subtilis*; however, in comparison, the region surrounding *dnaG *and *sigA *in *S. pyogenes *was completely divergent. It was noted that the 5' gene in the *E. coli MMSO, rpsU*, is at most ~15 kb upstream of each gram-positive MMSO suggesting a linkage between *rpsU, dnaG*, and *sigA *in gram-positive and gram-negative species. Of the genes immediately upstream of *dnaG*, it was found that *S. epidermidis *does not have a *yqxD *orthologue as found in *B. subtilis *and *L. monocytogenes *(*Lmof2365_1475*). *yqxD *and *Lmof2365_1475 *share 48% amino acid identity [17]. Just upstream of *dnaG *in *S. epidermidis *were two ORFs, *serp1129 *and *serp1130*. An ortholog of *serp1129 *is found upstream of *yqxD *and *Lmof2365_1475 *in *B. subtilis *(*yqfL*) and *L. monocytogenes *(*Lmof2365_1476*), respectively. Only *B. subtilis *has a *serp1130 *ortholog (*yqzB*). Bioinformatic analyses of *serp1129*, annotated as a hypothetical protein, shared 59% and 47% amino acid identity with *yqfL *(*B. subtilis*) and *Lmof2365_1476 *(*L. monocytogenes*), respectively. In addition, *serp1130*, annotated as a hypothetical protein containing a CBS domain, shared 59% amino acid identity with *B. subtilis yqzB*. These results suggest a strong conservation of the linkage between *dnaG *and *sigA *among the gram-positive genomes; however, the presence of a *serp1129 *ortholog upstream of *dnaG *in three of the four species appeared equally significant.

### Transcriptional analysis of the *S. epidermidis *MMSO

**Figure 2:**
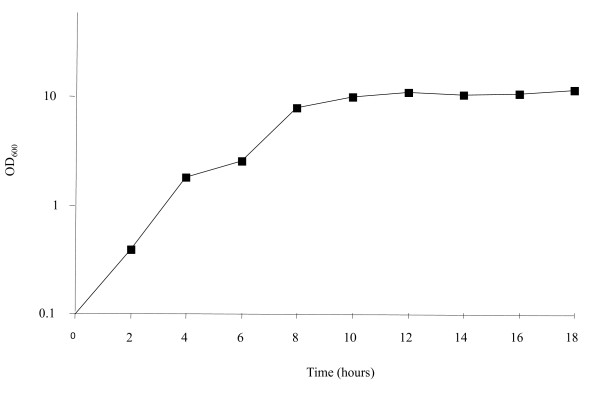
**Growth analysis of *S. epidermidis *1457**. *S. epidermidis *was grown aerobically in tryptic soy broth over a 18 hour time period. Growth was assessed by measuring the optical density at 600 nm.

**Figure 3:**
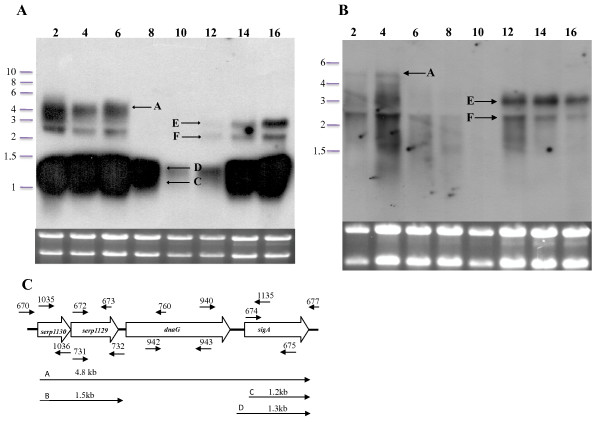
**Northern blot analysis of the *S. epidermidis *MMSO using a *sigA *and *dnaG *DNA probe**. The number above each lane in panels A (hybridized with a *sigA *probe) and B (hybridized with a *dnaG *probe) represents the time in hours of growth before each RNA sample was processed. A picture of the ethidium bromide stained gel is shown beneath each blot to serve as a loading control and verify RNA integrity. Arrows in panels A and B denote transcripts A, C through F as discussed in text. Panel C: Schematic depiction of the *S. epidermidis *MMSO. Small arrows above and below the schematic represent primer sets used in RT-PCR reactions and other cloning experiments. Arrows below the schematic correspond to transcripts A, B, C, and D as discussed in text.

A series of northern blots were performed to determine the number of transcripts and genes associated with the MMSO of *S. epidermidis. S. epidermidis *1457 was grown over a 18-hour period (Figure 2) and aliquots were taken at two-hour intervals for RNA extraction. The *sigA *DNA probe hybridized to five bands (labeled A, C-F; Figure 3A) of sizes 4.8 kb (band A), 1.3 kb (band D), 1.2 kb (band C), 3.0 kb (band E) and 2.5 kb (band F). Bands A, C-F were detected through six hours of growth (exponential growth phase) using a *sigA *probe; however, the largest transcript (band A) was not detected after six hours of growth. Bands E and F were detected again at 12 hours of growth (post-exponential phase). Bands C and D were variably expressed throughout the growth phase. The lack of detection of bands A, E and F in hours 8-10 corresponds to the shift from exponential to post-exponential phase growth (Figure 2). A similar banding pattern was observed when *dnaG *was used as a probe (Figure 3B). Transcripts correlating to band A were not detected with the *dnaG *probe after four hours of growth, whereas both mRNAs correlating to bands E and F were again detected in post-exponential growth (12-16 hours). However, bands C and D (Figure 3A) were not detected using *dnaG *as a probe, suggesting that both of these transcripts were comprised of *sigA *alone. A series of RT-PCR reactions were performed to determine the 5' and 3' ORF's encompassed within the *S. epidermidis *MMSO (data not shown). Appropriately sized products were amplified from *S. epidermidis *mRNA isolated during exponential phase when the following primer pairs were used: 1035 and 673; 672 and 760; and 940 and 1135 (primer pairs shown in Figure 3C). However, no amplicon was detected using primers 674/677 and 673/670. These data demonstrated *sigA *comprised the 3' end gene of the *S. epidermidis *MMSO whereas *serp1130 *was located at the 5' end.

**Figure 4:**
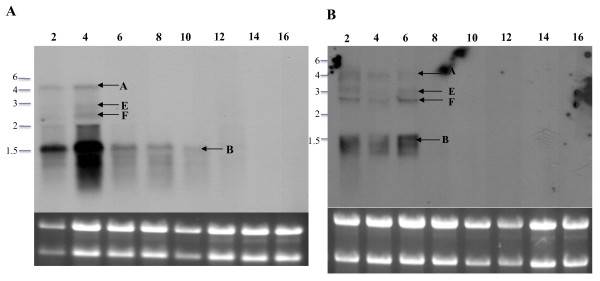
**Northern blot analysis of the *S. epidermidis *MMSO using a *serp1129 *and *serp1130 *DNA probe**. The number above each lane in panels A (hybridized with a *serp1129 *DNA probe) and B (hybridized with a *serp1130 *DNA probe) represents the time in hours of growth before each RNA sample was processed. A picture of the ethidium bromide stained gel is shown beneath each blot to serve as a loading control and verify RNA integrity. Arrows in panels A and B denote transcripts A, B, E and F as discussed in text.

To evaluate the transcriptional regulation of the 5' genes in the MMSO during *S. epidermidis *growth, *serp1129 *and *serp1130 *were used as probes in northern blot analyses (Figures 4A-B). Both of these probes hybridized to mRNA in a similar manner and identified four bands (A, B, E, and F). Bands A, E, and F were 4.8 kb, 3.0 kb, and 2.5 kb in size, respectively, and corresponded to the same bands of similar size when both *sigA *and *dnaG *were used as probes (Figures 3A-B). A unique 1.5 kb band (band B; Figure 4A-B) was detected with both probes. Since the length of *serp1129 *and *serp1130 *combined is 1319 bp, these data suggested that both *serp1129 *and *serp1130 *were encoded on one mRNA transcript. The transcripts associated with bands A and B were detected only in aliquots taken during the exponential growth phase.

Collectively, these data suggested the following: 1) the 4.8 kb (band A) transcript encompasses all four ORF's (*serp1129, serp1130, dnaG*, and *sigA*) and this transcript is detectable only when the cells are in exponential phase; 2) *sigA *is encoded within two unique transcripts (bands C and D) that are highly expressed throughout the growth phase (although expression is downregulated at 10-12 hours of growth); 3) *serp1129 *and *serp1130 *are encoded on a separate transcript (band B; or, alternatively, transcript A is prematurely terminated) that is expressed only in exponential phase; and 4) based on the size of transcripts E and F (3.0 kb and 2.5 kb, respectively), the size of the entire MMSO operon (4.8 kb), and the fact that all four probes hybridized to bands E and F, we could not determine the most probable location of these transcripts.

### Identification of transcriptional start sites

**Figure 5:**
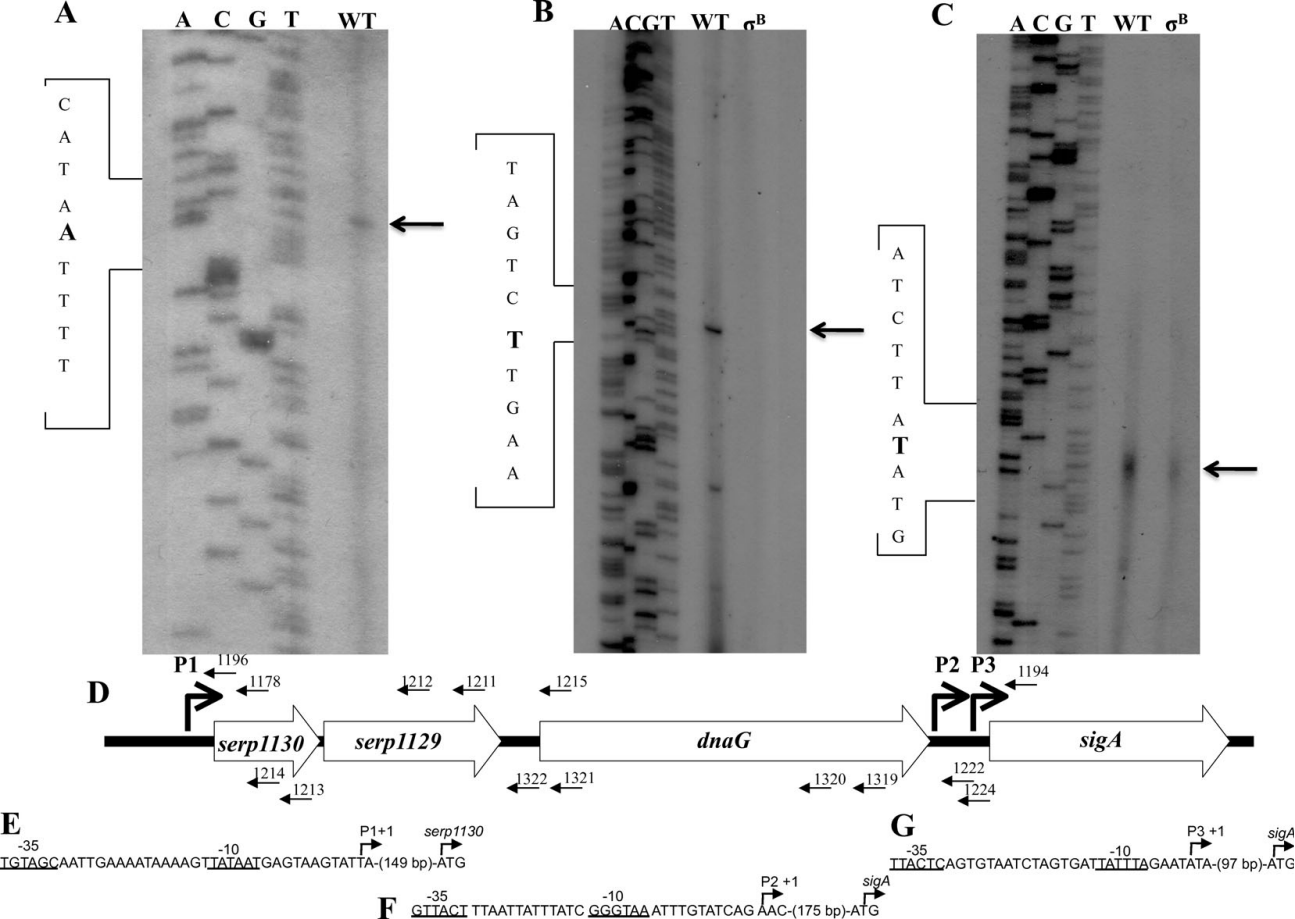
**Primer extension analysis of the *S. epidermidis *MMSO**. Primer extension showing the +1 transcriptional start site (denoted by small arrow) of the (A) P1 promoter upstream of *serp1130 *using primer 1178, (B) σ^B^-dependent P2 promoter upstream of *sigA *using primer 1222, and (C) P3 promoter upstream of *sigA *using primer 1194. WT above each panel represents wildtype *S. epidermidis *1457, whereas σ^B^denotes 1457 *sigB::dhfr*. (D) Schematic diagram showing the position of proposed promoters (P1, P2, and P3) in the MMSO of *S. epidermidis*. Small arrows depict the position of the primer extension and RACE primers used to detect the three transcriptional initiation sites. Sequence of putative -35 and -10 boxes, defined transcriptional start site (+1) and ATG start site of (E) P1 promoter, (F) σ^B^-dependent P2 promoter, and (G) P3 promoter.

Primer extension was performed to confirm the results of the northern blot analyses and to detect the transcriptional start site of the predicted transcripts shown in Figure 3C. Using mRNA collected after two hours of growth and primers 1178 and 1196 (Table 1 and Figure 5D), it was determined that the +1 site of transcript A was an adenine 152 bp upstream from the *serp1130 *ORF (Figure 5A) and was labeled as P1 in Figure 5D. No other additional transcript was detected in this 5' region of the MMSO suggesting that transcript B represents a prematurely terminated transcript A. Next, RNA isolated from aliquots taken during post-exponential phase (14 hours) was used to determine the +1 sites of transcripts C and D proximal to *sigA*. Using primers 1194 and 1224 (Table 1 and Figure 5D), two separate transcripts were identified. One +1 site (transcript D; Figure 3C) corresponded to a thymine 177 bp upstream from the *sigA *start codon (Figure 5B; P2 in Figure 5D), while the second +1 site (transcript C; Figure 3C) originated at a thymine 78 bp upstream of *sigA *(Figure 5C; P3 in Figure 5D).

Since the location of the +1 sites for transcripts E and F within the MMSO could not be predicted by northern blot analysis, several different primers were used in primer extension and RACE analysis. Although five primers were tested in primer extension analysis (1211, 1212, 1213, 1214, and 1215; Figure 5D) and four primers (1319, 1320, 1321, and 1322; Figure 5D) were utilized in 5' RACE analysis with RNA isolated from both exponential and stationary phase cultures, the +1 transcriptional start sites for the transcripts that corresponded to bands E or F could not be determined. To investigate whether transcripts E and F represented anti-sense RNA (to which the double stranded DNA probe would hybridize), both sense and anti-sense *sigA *RNA probes were constructed. Using RNA isolated at 4 and 16 hours, northern blot analyses demonstrated that the *sigA *anti-sense RNA probe detected the same transcripts as the DNA probe including transcripts A, B, C, D, E, and F (data not shown). However, the sense *sigA *RNA probe only hybridized weakly to the 16S and 23S rRNA bands (data not shown). Therefore, since all four probes (*serp1129, serp1130, dnaG*, and *sigA*) did not consistently detect transcripts E and F throughout the growth phase (Figures 3 and 4), transcripts E and F most likely represent processed or degraded forms of transcript A (4.8 kb).

### Transcription of *sigA *occurs from both σ^A^- and σ^B^-dependent promoters

**Figure 6:**
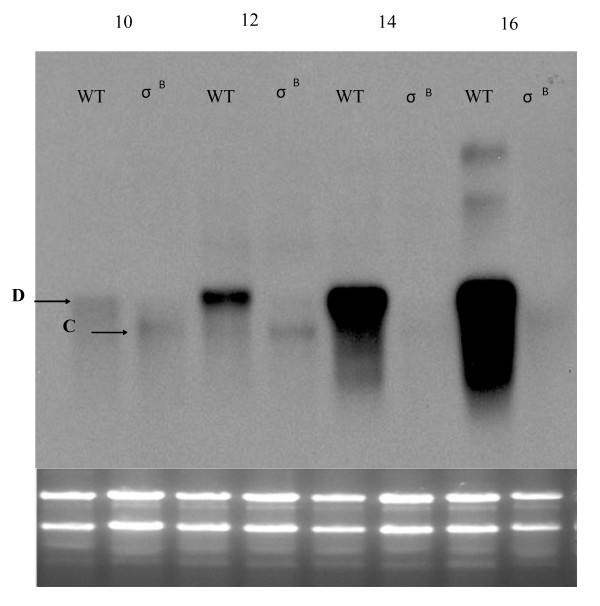
**Northern blot analysis of 1457 and 1457 *sigB::dhfr *using a *sigA *probe**. The number above each lane represents the time in hours of growth before each RNA sample was processed. WT above each lane represents wildtype *S. epidermidis *1457, whereas σ^B^denotes 1457 *sigB::dhfr*. Small arrows denote transcripts C and D as discussed in text.

Previous studies of the *E. coli *MMSO have shown the presence of a heat shock inducible promoter located directly upstream of the *sigA *ORF inside of the *dnaG *coding sequence [18]. A similar promoter has been identified within the *B. subtilis *MMSO [9]. To determine whether transcripts in the *S. epidermidis *MMSO originated from a σ^B ^promoter, RNA extracts from both wild type 1457 and 1457 *sigB::dhfr *were probed with *sigA *and *serp1129*. The northern analysis demonstrated no difference between 1457 and *1457 sigB::dhfr *RNA when probed with *serp1129 *(data not shown). However, transcript D was not detected in the 1457 *sigB::dhfr *RNA when *sigA *was used as a probe (Figure 6) suggesting *sigA*, the gene encoding the primary sigma factor used in staphylococci, is also transcribed from a σ^B^promoter. To confirm this northern blot result, a series of primer extension reactions were performed. Results showed that a P2 +1 site was not detected in RNA isolated from 1457 *sigB::dhfr *(Figure 5B), whereas the P3 +1 site was detected in both 1457 and *1457 sigB::dhfr *(Figure 5C). Putative -35 and -10 regions and the transcriptional start site of each promoter P1, P2, and P3 are shown in Figures 5E, F and 5G. The σ^B^-consensus sequence GttTww-_12-15_-gGgwAw was used to identify the putative σ^B^-P2 promoter sequence [11,19,20].

### Expression of Serp1129 in *S. epidermidis *1457

**Figure 7:**
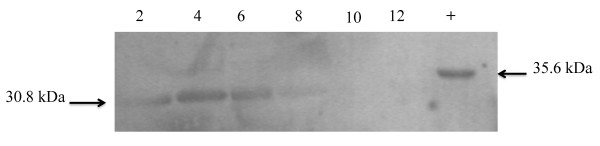
**Western blot analysis to demonstrate Serp1129 expression**. Western blot analysis showing the expression of Serp1129 from 2 to 12 hours of growth. Number above each lane represents the hour (growth) at which the protein sample was collected. The arrow on the left of the figure notes the expression of the 30.8 kDa native Serp1129 throughout growth of *S. epidermidis *1457. The "+" lane is the positive control containing the 35.6 kDa recombinant His- tagged Serp1129 protein and is denoted by an arrow on the right.

Since *serp1129 *was contained within the *S. epidermidis *MMSO and conserved in three of the four gram-positive genomes analyzed, expression and functional studies were performed. Anti-Serp1129 antibody was used in western blot studies to determine if Serp1129 was maximally produced during exponential growth as predicted by transcriptional analysis. Total protein samples from *S. epidermidis *1457 were taken every two hours from 2-12 hours of growth. These data demonstrated that *Serp1129 *was expressed at low levels at 2 hours and increased to the maximum level at 4 and 6 hours, and began to decrease at 8 hours with no Serp1129 being detected at the 10 or 12 hour time point (Figure 7). These data demonstrate that serp1129 transcript was translated, and that Serp1129 was only expressed in the exponential phase of growth as predicted by the previous northern blot analyses.

### Serp1129 is an ATP/GTP Binding protein

**Figure 8:**
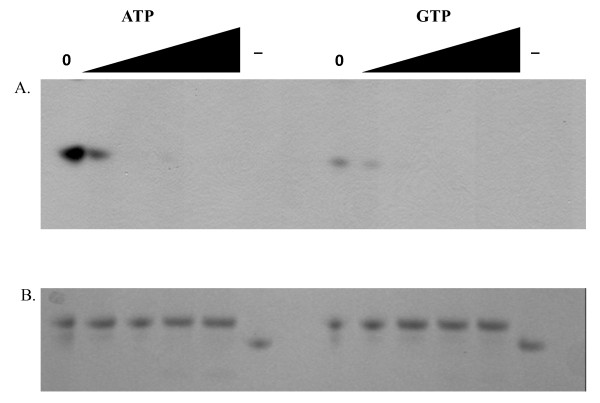
**ATP and GTP Competition Assays for Serp1129**. (**A**) ATP and GTP binding assay. The lane marked "0" indicates that no unlabeled ATP or GTP was added to the reaction and increasing levels (5, 10, 20, and 30 μM) of unlabeled ATP or GTP are indicated by the triangle above the appropriate lanes. The lanes marked as "-" are the negative control containing CidA [38], which does not bind ATP or GTP. **B**. SDS-PAGE loaded with the same protein concentration of Serp1129 as in Figure 6A and stained with Coomassie Blue; shown as a loading control.

The potential functional role of Serp1129 in *S. epidermidis *was further investigated as bioinformatic analyses indicated that Serp1129 shared 54% amino acid identity with *B. thuringiensis *ATCC 35646 RBTH_03589, a protein annotated as having an ATP/GTP binding motif. Recombinant Serp1129 was tested for the ability to bind ATP or GTP, and found both nucleotide analogs were able to bind Serp1129 (data not shown). Adding 5, 10, 20, and 30 μM of unlabeled ATP to the reaction mixture evaluated the specificity of ATP binding to recombinant Serp1129. The addition of 5 μM unlabeled ATP decreased the binding of labeled ATP to Serp1129, while no band was detected when 10 μM unlabeled ATP was added (Figure 8A). These data suggest that the unlabeled ATP was able to compete for the same binding site within Serp1129. A similar pattern was observed when GTP binding reactions were performed, however, less GTP was bound by Serp1129 as compared to ATP. A Coomassie Blue stained gel was loaded with an equivalent amount of protein used in the experiment and is shown as a loading control (Figure 8B). These results indicate that Serp1129 has an ability to bind both ATP and GTP but has a higher affinity for ATP.

## Discussion

*S. epidermidis *is a component of the normal skin flora of humans and yet is a significant cause of catheter and other biomaterial-related infections. This opportunistic pathogen is typically resistant to multiple classes of antibiotics. New targets, including those factors involved in DNA replication (e.g. primase), are needed for development of next generation antimicrobials. In the studies described here, *S. epidermidis *was used as a model organism to ascertain the transcriptional regulation of genes pertinent to DNA replication. Since it had not been previously described, it was necessary to characterize in detail the transcriptional regulation of the MMSO (containing *dnaG*) in *S. epidermidis*.

Several important differences were identified between the MMSO of *S. epidermidis *and the previously well characterized *B. subtilis *MMSO [8,9,21]. The *S. epidermidis *MMSO contained two genes not previously recognized as part of a MMSO; *serp1130 *and *serp1129*. Both genes encode for proteins with unknown functions. Bioinformatic analysis of the amino acid sequence of Serp1129 demonstrated that it possessed an ATP or ATP-derivative binding motif while Serp1130 contained a CBS (cysteine β-synthase) domain, a motif frequently identified in human proteins [22-24]. Second, the *B. subtilis *MMSO is known to have 7 distinct transcription initiation sites, whereas only three transcriptional start sites and six transcripts were detected in the *S. epidermidis *MMSO [9]. Although speculative, the greater complexity of the transcriptional regulation of the MMSO in *B. subtilis *in comparison to *S. epidermidis *may be due to the regulation of the sporulation cascade [25]. One transcription initiation site was identified at the 5' end of the MMSO and two were identified at the 3' end initiating *sigA *transcription. It is probable that both transcripts A and B originate from the same transcription initiation site at the 5' end of the MMSO and that transcript B is prematurely terminated at the 3' end of *serp1129 *(Figure 3C), especially since a rho-independent termination site exists between *rpsU *and *dnaG *in a large number of gram-negative MMSOs [2]. Western blot analysis demonstrated that Serp1129 was maximally detected in exponential phase growth, in agreement with the transcriptional analysis of the *serp1129 *expression.

Our study found that the primary sigma factor of *S. epidermidis, sigA*, [26] is transcribed from two promoters, one of which is σ^B^-dependent. Currently, the model for bacterial sigma factor exchange does not account for transcriptional differences between each sigma factor. The model only examines competition between the free sigma factor pool for RNA polymerase [27-29]. Therefore, the sigma factor pool that is in excess will bind to RNA polymerase resulting in the transcription of a subset of genes [27,28]. However, within *B. subtilis*, σ^B ^has a 60-fold lower affinity for RNA polymerase than σ^A ^suggesting other layers of regulation may exist to ensure sigma factor exchange [29]. Therefore, transcriptional regulation of sigma factor genes may act as a second layer of sigma factor regulation which functions to fine-tune the free sigma factor pool, ultimately affecting sigma factor exchange with RNA polymerase. We hypothesize that the presence of a σ^B^-dependent promoter upstream of *sigA *ensures that relevant concentrations of σ^A ^are present under all metabolic conditions to interact with RNA polymerase, even under conditions of cellular stress (i.e. σ^B ^activation). In addition, σ^B ^regulation of *sigA *may also be important for the return of the bacterium from a stress response to a normal transcriptional pattern. In support of this concept, recent studies in *Synechocystis *PCC66803 demonstrated that induction of one sigma factor altered the transcription of the remaining sigma factors suggesting a transcriptional cross-talk within the sigma factor system [30].

In addition to transcripts A (4.8 kb), B (1.5 kb), C (1.2 kb) and D (1.3 kb), two transcripts E and F were detected using all four probes. However, a transcriptional initiation site for transcripts E and F was not identified using two separate methodologies, specifically primer extension and 5' RACE. Therefore, we propose that transcripts E and F are processed/degraded products of the larger 4.9 kb transcript A. It is known that the MMSO of *E. coli *is selectively cleaved by RNaseE [31]; providing additional evidence that transcripts E and F could represent a regulated, processed form of transcript A. It is possible, although their sizes are indistinguishable, that transcripts E and F detected in exponential phase are unique from that detected in late exponential or stationary phase (i.e. 12-16 hours of growth; Figures 3A-B). However, a potential +1 site for transcripts E and F was not detected using total RNA isolated from both exponential and stationary phase cultures. Clearly, further experimentation is needed to determine the origin and function of transcripts E and F.

The conservation of Serp1129 orthologs in three gram-positive species led us to investigate the potential functional role of Serp1129. ATP and GTP binding assays showed that Serp1129 was capable of binding ATP and GTP. Studies of *Streptomyces *NrdR have shown that the binding of ATP or dATP into a pocket within the protein affect its ability to bind and act as a transcriptional regulator of the ribonucleotide reductases genes [32]. However, it is unknown whether the ability of Serp1129 to bind ATP or GTP functions in regulating transcription of the MMSO during exponential growth.

Serp1130 may also play a pivotal role in sensing the energy status of the cell and regulation of replication proteins within *S. epidermidis*. CBS domains are necessary for the energy sensing mechanism in some proteins such as AMP-activated protein kinase (AMPK) [24,33,34]. Recent data from studies in bacteria have demonstrated that the CBS domain within YrbH of *Yersinia pestis *negatively regulates the organisms ability to produce biofilm by responding to ATP concentrations within the cell [35]. In addition, a CBS domain within *Lactococcus lactis *OpuA is involved in sensing ionic strength [36,37]. It follows that a protein with the ability to sense environmental stress or the energy status of the cell could be a significant regulator of DNA replication. Our laboratory is currently investigating whether *serp1129 *and *serp1130 *are involved in the transcriptional regulation of the MMSO and/or other replication genes.

## Conclusions

These studies demonstrated that the *S. epidermidis *MMSO contains two previously unidentified ORFs (*serp1129 *and *serp1130*) and that *sigA *transcription is regulated by a σ^β ^promoter. The transcriptional regulation of *sigA *by σ^B ^suggests that the staphylococcal σ^B ^regulon is regulated at both the transcriptional and post-transcriptional levels. Further assays demonstrated that Serp1129 is an ATP/GTP binding protein; its connection to other functions found within genes encoded by the MMSO is unknown. Finally, although *sigA *was actively transcribed in both the exponential and post-exponential phases of growth, *serp1130, serp1129 *and *dnaG *were most transcriptionally active during exponential growth. We are currently testing the hypothesis that genes involved in DNA replication, including the MMSO, are co-regulated in the exponential growth phase through a common regulator or metabolite.

## Authors' contributions

KB performed all the molecular genetic experiments, drafted the manuscript and participated in the design of the experiments. LK participated in the northern blot experiments. ML participated in the design and implementation of the protein expression studies and ATP/GTP binding assays. SH and PF coordinated all aspects and design of the study. All authors read and approved the final manuscript.
